# The International Medical Annual

**Published:** 1902-09

**Authors:** 


The International Medical Annual. A Year Book of Treatment and Practitioner's Index. Nineteenth year. 1901. Pages, 681. Price, 3.00. New York: E. B. Treat & Co., 241 West 23d St.; Chicago, 109 Clark St.

The same general plans have been adopted in the preparation of this volume that have proved popular in former editions. One notes with particular interest the articles on toxins and antitoxins, and on the "light treatment."

A special article on "X-Ray Treatment," by Macintyre, of Glascow, is timely.

The work has summed up the year's treatment of disease in a most pleasing and practical manner. T. J. B.

